# XANNpred: Neural nets that predict the propensity of a protein to yield diffraction-quality crystals

**DOI:** 10.1002/prot.22914

**Published:** 2010-10-19

**Authors:** Ian M Overton, C A Johannes van Niekerk, Geoffrey J Barton

**Affiliations:** 1 School of Life Sciences Research, College of Life Sciences, University of DundeeDundee, DD1 5EH, United Kingdom; 2 MRC Human Genetics Unit, Institute of Genetics and Molecular Medicine, Western General HospitalEdinburgh, EH4 2XU, United Kingdom

**Keywords:** computational biology, bioinformatics, crystallization, software, artificial neural network, predictor

## Abstract

Production of diffracting crystals is a critical step in determining the three-dimensional structure of a protein by X-ray crystallography. Computational techniques to rank proteins by their propensity to yield diffraction-quality crystals can improve efficiency in obtaining structural data by guiding both protein selection and construct design. XANNpred comprises a pair of artificial neural networks that each predict the propensity of a selected protein sequence to produce diffraction-quality crystals by current structural biology techniques. Blind tests show XANNpred has accuracy and Matthews correlation values ranging from 75% to 81% and 0.50 to 0.63 respectively; values of area under the receiver operator characteristic (ROC) curve range from 0.81 to 0.88. On blind test data XANNpred outperforms the other available algorithms XtalPred, PXS, OB-Score, and ParCrys. XANNpred also guides construct design by presenting graphs of predicted propensity for diffraction-quality crystals against residue sequence position. The XANNpred-SG algorithm is likely to be most useful to target selection in structural genomics consortia, while the XANNpred-PDB algorithm is more suited to the general structural biology community. XANNpred predictions that include sliding window graphs are freely available from http://www.compbio.dundee.ac.uk/xannpred Proteins 2011. © 2010 Wiley-Liss, Inc.

## INTRODUCTION

Substantial global efforts have been focused on the large-scale structural characterization of proteomes (see http://www.isgo.org/home/index.php and Refs.^1^–^5^). However, the high-throughput approaches of “structural genomics” (SG) consortia typically result in high-resolution molecular models for only 5% to 10% of selected protein targets.^4^,^6^,^7^ Various strategies have been proposed to increase this rate of success, such as obtaining one representative structure per protein family and working with multiple orthologues.^8^–^12^ In order to realize the potential of these approaches, it is necessary to rank proteins according to their propensity to make good progress through the structure determination pipeline. Crystallization is a bottleneck in structure determination so one approach is to estimate the likelihood of obtaining diffraction-quality crystals as part of the target selection process.^13^–^16^

Studies of the relationship between protein sequence properties (hydrophobicity, charge, etc.) and progression through the structure determination pipeline have suggested features relevant to predicting crystallization propensity.^16^–^18^ Several predictors have been developed in this area including the OB-Score,^19^ XtalPred,^20^ ParCrys,^21^ and PXS.^16^ These methods draw on a variety of computational techniques, training data, and protein sequence properties. While some studies have examined the biophysical mechanisms underlying protein sequence determinants of crystallization propensity,^16^,^18^,^22^ the work presented here focuses on predicting protein targets' propensity to progress to the stage of diffraction-quality crystals.

This paper describes two new neural networks (XANNpred-PDB and XANNpred-SG) that predict protein propensity to yield diffraction-quality crystals. In addition, a sliding window of XANNpred scores along the length of individual protein sequences provides a guide for selection of regions most likely to succeed in structural studies.

## METHODS

### Datasets summary

The selection of training and testing data is a critical stage in the development and evaluation of a predictive algorithm. Selection of inappropriate data can lead to unrealistic estimates of an algorithm's performance, and may bias the algorithm toward only a subset of possible problems. Therefore, rigorous procedures were applied in selecting datasets for the development and testing of the XANNpred predictors. These datasets are detailed in Supporting Information, Figure S1, Table S1 and described in the sections below. In summary, data to represent proteins that produce diffraction-quality crystals were taken from either PDB^23^ or PepcDB (http://pepcdb.pdb.org/index.html) and these were respectively taken as the positive training (and testing) sets for the XANNpred-PDB and XANNpred-SG predictors. Negative data for both XANNpred-PDB and XANNpred-SG were protein targets where work was stopped before obtaining crystals as reported in PepcDB. PepcDB provides details of construct sequences and reasons for stopping work, while the PDB is less influenced than PepcDB by the sequence-based target selection criteria of Structural Genomics consortia. Therefore PDB and PepcDB provide complementary data sources. In order to produce representative datasets for algorithm development and evaluation, a stringent redundancy filtering procedure was applied. This procedure aims to generate a set of sequence and structurally dissimilar proteins, in order to minimize bias and to control for overlap in the training and blind test datasets.^24^ Blind test datasets were not used in any stage of algorithm development, as an essential condition for fair assessment of predictive performance.^24^

### Production of training and blind test datasets

The protocols to generate datasets for XANNpred-PDB were as follows. In order to obtain representatives of diffraction-quality crystals, the 1538 SCOP 1.69 superfamily representatives^25^,^26^ were searched against the PDB with BLASTP,^27^ to identify the top-scoring PDB sequence for each superfamily representative. After exclusion of NMR structures, this gave the PDB_TOP dataset (1180 sequences) which was structural superfamily non-redundant. To provide sequence redundancy filtering PDB_TOP was combined with SEG^28^ and helixfilt (D. Jones, personal communication) filtered sequences from UniRef50^29^ to give the database PDB_TOP_U50. Searching PDB_TOP against PDB_TOP_U50 with PSIBLAST^27^ followed by single-linkage clustering according to published thresholds^30^ gave the PDB_CLUS dataset. Further clustering with AMPS^31^ SD score threshold of 5 and exclusion of structures with resolution >3Å provided a second, stringent sequence redundancy filtering step to generate the PDB_POOL dataset of 888 nonredundant sequences. Sequences where work had been stopped before crystals were obtained were represented by PepcDB (http://pepcdb.pdb.org/index.html) trial sequences with Status “work stopped” and Status History including “Cloned” but without an indicator of crystallisation (e.g. “Crystals”). Sequences were excluded if they were DNA, or annotated as “test target,” or where the stopDetails included “duplicate target found,” thus generating PEP_WS. A PSIBLAST filtering step of PEP_WS against a database of the whole PDB embedded in UniRef50 was performed using published thresholds.^30^ This filtering step was implemented because structural genomics consortia deselect targets that match to solved structures.^9^ Therefore some of the “work stopped” sequences are associated with solved structures and so should be excluded from the negative dataset. The remaining sequences were clustered with a PSIBLAST all-versus-all search as described for PDB_POOL, to generate PEP_CLUS as a first step in removing sequence redundancy. A HMMER search^32^,^33^ of PEP_CLUS against Pfam was applied to select a representative PEP_CLUS sequence for each of the 807 Pfam profiles matched, to generate PEP_PFAM (*E*-value threshold 0.1, topscoring match taken). Redundancy filtering with HMMER/Pfam is complementary to the PSIBLAST-based filtering and provides for more sensitive detection of evolutionary relationships. As a final, stringent sequence redundancy filtering step PEP_PFAM was clustered with AMPS^31^ at SD score threshold of 5 to produce a set of 747 nonredundant sequences (PEP_NEG). The above redundancy filtering approaches, involving three different algorithms, represents a highly stringent protocol that controls for overlap in the training and blind test datasets as prerequisite for proper evaluation of the XANNpred algorithms.

For the XANNpred-SG algorithm a second positive dataset was taken from PepcDB (http://pepcdb.pdb.org/index.html) trial sequences with Status History including “diffraction-quality crystals” (PEP_DIFF, 36,156 sequences). PEP_DIFF was processed according to the protocol described in generating PEP_NEG but omitting the PDB filtering step, to produce a set of 521 nonredundant sequences (PEP_POS). Negative data for the XANNpred-SG algorithm was taken from the PEP_NEG dataset.

In order to generate balanced datasets for training and testing the XANNpred-PDB algorithm, 747 sequences (PDB_POS) were randomly chosen from PDB_POOL to balance with the 747 sequences in PEP_NEG. A random selection of 75 sequences from each of PDB_POS and PEP_NEG were set aside as the blind test set (TEST-PDB, 150 sequences). The remaining 672 sequences from each of PDB_POS and PEP_NEG (POS_TRAIN-PDB and NEG_TRAIN-PDB respectively) were combined to form the XANNpred-PDB training dataset (TRAIN-PDB, 1344 sequences), which was input for 10-fold cross-validation. Balanced datasets for training and testing the XANNpred-SG algorithm were generated from PEP_POS and PEP_NEG in a similar fashion (details given in Supp. Info.).

### Production of hybrid blind test datasets

Datasets were constructed in order to investigate the algorithm robustness to predicting over proteins from databases that were not used in algorithm development. These datasets therefore offer a more stringent evaluation of the algorithms because they aim to control for bias inherent across individual databases. XANNpred-PDB was initially developed and tested with PDB sequences to represent diffraction-quality crystals; therefore the XANNpred-PDB hybrid blind test dataset took sequences from PepcDB in place of the PDB sequences. Conversely, XANNpred-SG was developed and tested with PepcDB sequences, and so the XANNpred-SG hybrid blind test dataset took PDB sequences as representatives of diffraction-quality crystals in place of PepcDB sequences. Stringent filtering procedures were applied to the hybrid test datasets, in order to control for overlap with the data used in algorithm development.

To generate a hybrid blind test set for XANNpred-PDB, sequences from the “diffraction-quality” portion of TEST-SG (POS_TEST-SG, 53 sequences) were searched against the XANNpred-PDB training data (TRAIN-PDB) with BLASTP.^27^ Matches were assigned with published thresholds,^30^ and matching sequences were excluded to give POS_TEST-SG_FILT (44 sequences). A random selection of 44 sequences from the “work stopped” portion of TEST-PDB produced NEG_TEST-PDB44. TEST-PDB was already a blind test dataset for XANNpred-PDB and therefore NEG_TEST-PDB44 did not require any further filtering to eliminate overlap with XANNpred-PDB training data. NEG_TEST-PDB44 was combined with POS_TEST-SG_FILT to form the HTEST-PDB dataset (88 sequences). A similar approach was applied to generate a hybrid blind test set for XANNpred-SG (details given in Supp. Info.).

### Features

The 428 features employed by XANNpred were: 20 amino acid and 400 dipeptide frequencies, isoelectric point, averaged GES hydrophobicity,^34^ fraction of strand and helix residues predicted by Jpred,^35^ fraction of RONN disorder,^36^ sequence length, fraction of TMHMM2 transmembrane regions,^37^ and molecular weight. The features and their scaled values are summarized in Supporting Information, Table S2. Feature selection was based on our expectations of sequence-derived properties that may be informative, according to previous studies.^9^,^13^,^17^,^18^,^38^–^40^

### The neural network

Two feed-forward artificial neural networks were created within the SNNS package^41^ named XANNpred-PDB and XANNpred-SG to reflect the different datasets employed in the development of these algorithms. The networks each had 428 input nodes, a single hidden layer with 100 nodes and 1 output node. The number of hidden nodes was not optimized, however an architecture with 100 hidden nodes was found to provide good performance in the JPRED algorithm.^35^ XANNpred-PDB and XANNpred-SG had respective optima for the number of training cycles at 2100 and 1600, performed using back-propagation with a learning rate of 0.01 and an “early stopping” protocol.^24^ Sequences from the positive and negative training sets had target outputs of 1 and 0, respectively. From cross-validation over the training data, the XANNpred-PDB/XANNpred-SG Area under the Receiver Operator Characteristic (AROC) curves were 0.784/0.823, respectively. The cutoffs for XANNpred-PDB and XANNpred-SG Artificial Neural Network output values were 0.517 and 0.418, respectively; and were chosen to maximize Matthews correlation coefficient (respective values 0.462, 0.525) over the training data.

### Sliding window system

In order to study the utility of XANNpred in identifying regions of a protein more likely to produce diffraction-quality crystals, the algorithm was applied to a sliding window of 61 amino acids rather than the entire protein sequence and the network outputs reported for the central amino acid. The window size was chosen to resemble the length of a relatively small domain, but was not optimised. The whole protein sequence was analyzed by relevant external programs (e.g. Jpred,^35^ TMHMM2^37^) and a sliding window of 61 residues was passed over the output from these programs. However, windowed values for amino acid and dipeptide frequencies as well as the pI, hydrophobicity, length and molecular weight features were calculated directly over the 61-residue window sequences. Feature values associated with each window position in the sequence were taken as input to the XANNpred-PDB artificial neural network. By this process a XANNpred score was assigned to each window position in the sequence. A graph of the XANNpred sliding window was visually inspected for each of the proteins in the NEG_TEST-PDB dataset.

## RESULTS AND DISCUSSION

**Figure 1:**
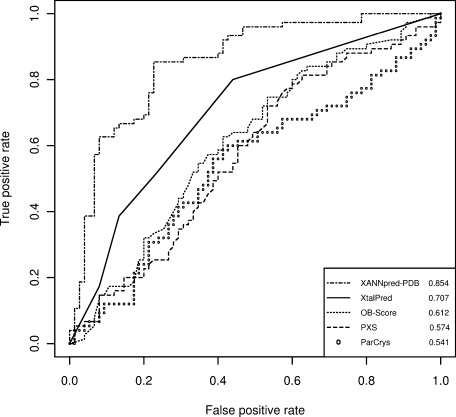
ROC curves for XANNpred-PDB, XtalPred,^20^ OB-Score,^19^ PXS,^16^ and ParCrys^21^ on the blind test dataset TEST-PDB. XANNpred-PDB significantly outperforms the next best algorithm XtalPred (two-tailed *P* ≤ 0.0062). Areas under the ROC curves are given in the bottom right-hand corner. This figure was generated using the R package.^42^

**Figure 2:**
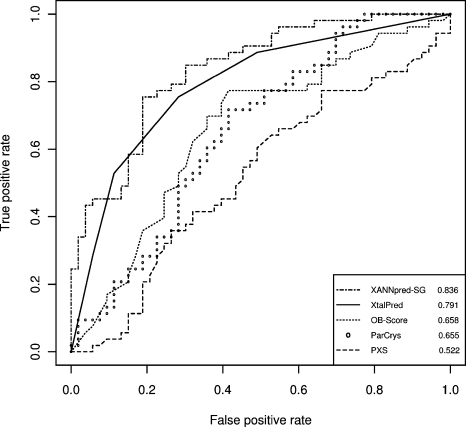
ROC curves for XANNpred-SG, XtalPred,^20^ OB-Score,^19^ ParCrys,^21^ and PXS^16^ on the blind test dataset TEST-SG. Areas under the ROC curves are given in the bottom right-hand corner. This figure was generated using the R package.^42^

Table I summarizes the performance of six algorithms (XANNpred-PDB, XANNpred-SG, XtalPred, ParCrys, OB-Score, PXS) on the blind test datasets. XANNpred-PDB accuracy and Matthews correlation values on the TEST-PDB dataset were 81.3% and 0.63, respectively. Figure 1 shows Receiver Operator Characteristic (ROC) curves for relevant algorithms predictions on the TEST-PDB dataset which was not used in feature selection, machine learning or any other aspect of XANNpred-PDB development. XANNpred-PDB had a significantly larger area under the ROC curve than the next best algorithm XtalPred (two-tailed *P* ≤ 0.0062). The maximum possible XtalPred accuracy and Matthews correlation on TEST-PDB were 68.0% and 0.37, respectively. The procedure to convert XtalPred classes into scores for ROC analysis is detailed in Supporting Information, section 3. The XANNpred-SG algorithm gave accuracy and Matthews correlation values of 75.5% and 0.52, respectively on the blind test dataset TEST-SG. Figure 2 shows ROC curves for predictions on TEST-SG; XANNpred-SG had a slightly larger area under the ROC curve than XtalPred. The maximum possible XtalPred accuracy and Matthews correlation on TEST-SG were 73.6% and 0.47, respectively.

**Table I:** Summary of Performance on Blind Test Datasets

	Dataset							
	TEST-PDB		TEST-SG		HTEST-PDB		HTEST-SG	
Algorithm	AROC	MCC	AROC	MCC	AROC	MCC	AROC	MCC
XANNpred-PDB	0.854	0.63	^—^a	^—^a	0.810	0.50	^—^a	^—^a
XANNpred-SG	^—^a	^—^a	0.836	0.52	^—^a	^—^a	0.877	0.58
XtalPred^b^	0.707	0.37 (0.29)	0.791	0.47 (0.47)	0.770	0.48 (0.48)	0.701	0.34 (0.27)
OB-Score^b^	0.612	0.23 (0.17)	0.658	0.37 (0.31)	0.644	0.32 (0.30)	0.613	0.24 (0.19)
ParCrys^b^	0.541	0.17 (0.12)	0.655	0.36 (0.25)	0.634	0.32 (0.21)	0.562	0.23 (0.13)
PXS^b^	0.574	0.21 (0.17)	0.522	0.13 (0.02)	0.599	0.30 (0.05)	0.416	0 (−0.02)

a These values may be inflated due to overlap with training data and therefore are omitted from the table. For completeness, respective AROC/MCC values for XANNpred-SG on TEST-PDB are 0.917/0.66; on HTEST-PDB 0.880/0.62. Respective AROC/MCC values for XANNpred-PDB on TEST-SG are 0.822/0.47; on HTEST-SG 0.857/0.65.

b Matthews correlation values given for XtalPred, OB-Score, ParCrys, and PXS are maximum possible values. Matthews correlation values in brackets were determined with predictive thresholds quoted in the literature for OB-Score and ParCrys; bracketed values for XtalPred reflect a threshold of 3; bracketed values for PXS reflect a threshold of 0.2.

Key data for training XtalPred^20^ and ParCrys^21^ were taken from SG consortia, so it is possible that XtalPred and ParCrys are optimized for SG datasets. It is routine for SG consortia to apply sequence-based selection constraints on their targets; these constraints influence the composition of databases such as PepcDB.^8^,^9^,^43^ Consistent with the idea that XtalPred and ParCrys are optimized for prediction over SG datasets, both XtalPred and ParCrys had larger areas under their ROC curve on TEST-SG compared with TEST-PDB; while these differences were not significant, the trend is suggestive. Moreover, XANNpred-PDB significantly outperforms XtalPred on TEST-PDB (two-tailed *P* ≤ 0.0062), while XANNpred-SG and XtalPred have similar performance on TEST-SG (as discussed in the preceding paragraph). Further investigations were made to determine whether XANNpred-PDB and XANNpred-SG predictions were respectively optimized to predict over the PDB and SG (PepcDB) datasets. For this purpose, hybrid blind test datasets were generated with positive (diffraction quality crystals) examples taken from an alternative source database (i.e. PDB/PepcDB). Therefore XANNpred-SG predictions were generated for a hybrid blind test dataset where positive examples were taken from the PDB (HTEST-SG); XANNpred-PDB predictions were generated for a hybrid blind test dataset where positive examples were taken from PepcDB (HTEST-PDB). A summary of all datasets is given in Supporting Information, Table S1. Both HTEST-SG and HTEST-PDB took negative examples from PepcDB and were controlled to be independent of the relevant training datasets. See Methods for more detailed discussion of the hybrid blind test datasets. Supporting Information, Figures S2 and S3 show the algorithms' performance on the HTEST-PDB and HTEST-SG datasets respectively. The results for XANNpred-SG on HTEST-SG were similar to those obtained on TEST-SG (ΔAROC two-tailed *P* ≤ 0.43); for XANNpred-PDB the results on HTEST-PDB were similar to those obtained over TEST-PDB (ΔAROC two-tailed *P* ≤ 0.43). Therefore both XANNpred-SG and XANNpred-PDB appeared robust to predicting on blind test datasets from either PDB or PepcDB. As shown in Table I XANNpred-PDB significantly outperformed XtalPred on TEST-PDB (ΔAROC two-tailed *P* ≤ 0.0062) while similar performance was found on HTEST-PDB (ΔAROC two-tailed *P* ≤ 0.56). Furthermore, XANNpred-SG significantly outperformed XtalPred on HTEST-SG (ΔAROC two-tailed *P* ≤ 0.007), with similar performance on TEST-SG (ΔAROC two-tailed *P* ≤ 0.45). Therefore both XANNpred-PDB and XANNpred-SG significantly outperformed XtalPred on data drawn from the PDB (TEST-PDB, HTEST-SG), while the XANNpred algorithms gave similar results to XtalPred on SG data (TEST-SG, HTEST-PDB). The PDB contains a number of membrane proteins, which are frequently excluded from structural genomics efforts and so expected to be under-represented in the PepcDB database. However the POS_TEST-PDB dataset only had one sequence (1.3%) with predicted transmembrane regions. Therefore the expected enrichment of membrane proteins in the PDB (when compared with PepcDB) is of minor importance in explaining the significantly better performance of both XANNpred-PDB and XANNpred-SG over XtalPred on PDB-based datasets. These results are consistent with the knowledge that XtalPred was trained on SG data.^20^ The analysis presented in this article makes a generous assessment of XtalPred performance, because the best possible values for XtalPred predictions were taken over the datasets. Also, XtalPred predictive power may be inflated due to the potential for overlap between these test data and the XtalPred training data. In summary, both XANNpred algorithms were robust to predicting over data from either PDB or SG consortia (PepcDB), and outperformed the other algorithms examined.

The OB-Score and ParCrys AROC on TEST-PDB were 0.612 and 0.541 respectively, although this difference was not significant (*P* ≤ 0.28). Also, OB-Score and ParCrys had similar AROC on TEST-SG (0.658, 0.655 respectively). In earlier work, ParCrys significantly outperformed the OB-Score over blind test datasets taken from TargetDB.^21^ These data suggest that the OB-Score may be more robust to differences in database composition than ParCrys. One explanation for these findings may be that while ParCrys has a more sophisticated statistical model and additional features compared with the OB-Score,^21^ selected ParCrys features reflect the TargetDB^44^ composition when ParCrys was trained.

The PXS algorithm performed relatively poorly over the data examined, which suggests that surface entropy may not be an overriding factor for the successful progression of selected targets to crystal structures. It is important to note that PXS was developed to predict the crystallization of “well behaved” soluble proteins,^16^ which is a different aim to the one that examined here; namely to predict the progression of a protein through the structure determination pipeline to the stage of diffraction-quality crystals. The XANNpred algorithms were developed to facilitate prioritization of proteins with the particular balance of properties required for success at all of the pipeline stages necessary for the production of diffracting crystals.

**Figure 3:**
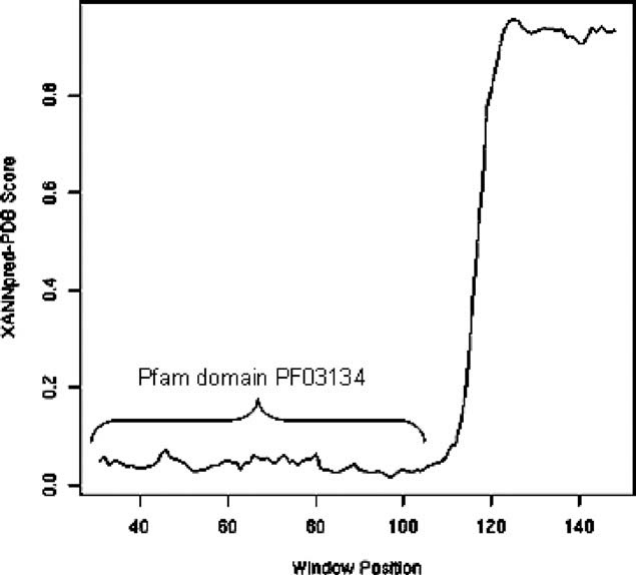
XANNpred-PDB sliding window plot for “HVA22-like protein a” (Q9S7V4). Residues 92 to 177 fall into windows with very high XANNpred score (>0.9), while the centre position of very high-scoring windows spans residues 123 to 148. The 85 residues within very high-scoring windows therefore offer a potentially promising starting point for work to crystallize the C-terminal region of “HVA22-like protein a.”

In order to investigate the variation of XANNpred score along the length of individual protein sequences, a sliding window system was implemented (methods). This approach is anticipated to have applications in construct design. Figure 3 shows a XANNpred-PDB score plot for the “HVA22-like protein a” from *Arabidopsis thaliana* (Q9S7V4), which was part of the NEG_TEST-PDB dataset. “HVA22-like protein a” was a selected structural genomics target annotated as “Work Stopped” in the PepcDB database (http://pepcdb.pdb.org/index.html). It is induced in response to stress (cold, drought, salt) and annotated with the Pfam domain PF03134.^33^,^45^ The proteins in this Pfam family include tumor suppressors deleted in severe human familial adenomatous polyopsis.^46^ The region of “HVA22-like protein a” that matched to the Pfam domain PF03134 had very low XANNpred score; however, the remainder of the protein was very high-scoring and so predicted to be relatively amenable to crystallization. This example provides indication of how the XANNpred sliding window plot may be helpful in construct design. Further experimental work would be required to validate this approach, which is beyond the scope of this study.

## CONCLUSIONS

XANNpred is a pair of artificial neural networks that may be used in structural biology protein target selection. From analysis of several nonredundant blind test datasets, XANNpred was found to outperform the other available algorithms in predicting the successful progression of a protein target through the experimental processes required to produce diffraction-quality protein crystals. However, XANNpred is not anticipated to be strongly predictive of transmembrane protein crystallization propensity. High XANNpred-SG scores predict that the protein would yield diffraction-quality crystals in a structural genomics pipeline. Therefore, XANNpred-SG is suggested to be most applicable to proteins that have passed structural genomics consortia selection criteria, and that are to be approached by “high-throughput” laboratory methods. The XANNpred-PDB scores predict crystallization success for the range of methodologies taken in producing PDB structures, including traditional laboratory methods; XANNpred-PDB is therefore expected to be more relevant to the structural biology community as a whole. XANNpred predictions, including sliding window graphs are freely available from http://www.compbio.dundee.ac.uk/xannpred. We would welcome suggestions of genomes or other large sequence sets for analysis by XANNpred.

## References

[1] Burley S, Almo S, Bonanno J, Capel M, Chance M, Gaasterland T, Lin D, Sali A, Studier FW, Swaminathan S (1999). Structural genomics: beyond the Human Genome Project. Nat Genet.

[2] Hol W (2000). Structural genomics for science and society. Nat Struct Biol.

[3] Stevens RC, Yokoyama S, Wilson IA (2001). Global efforts in structural genomics. Science.

[4] Service R (2002). Tapping DNA for structures produces a trickle. Science.

[5] Chandonia J-M, Brenner SE (2006). The impact of structural genomics: expectations and outcomes. Science.

[6] Terwillinger TC (2000). Structural genomics in North America. Nat Struct Biol.

[7] Service R (2005). Structural biology: structural genomics. Round 2. Science.

[8] Chandonia JM, Brenner SE (2005). Implications of structural genomics target selection strategies: Pfam5000. Whole genome, and random approaches. Proteins.

[9] Brenner SE (2000). Target selection for structural genomics. Nat Struct Biol.

[10] Hiu R, Edwards E (2003). High-throughput protein crystallisation. J Struct Biol.

[11] Liu J, Hegyi H, Acton TB, Montelione GT, Rost B (2004). Automatic target selection for structural genomics on eukaryotes. Proteins.

[12] Savchenko A, Yee A, Khachatryan A, Skarina T, Evdokimova E, Pavlova M, Semesi A, Northey J, Beasley S, Lan N, Das R, Gerstein M, Arrowmith C, Edwards A (2003). Strategies for structural proteomics of prokaryotes: quantifying the advantages of studying orthologous proteins and of using both NMR and X-ray crystallography approaches. Proteins.

[13] Pusey ML, Liu Z-J, Tempel W, Praissman J, Lin D, Wang B-C, Gavira JA, Ng JD (2005). Life in the fast lane for protein crystallization and X-ray crystallography. Progr Biophys Mol Biol.

[14] Chayen NE (2004). Turning protein crystallisation from an art into a science. Curr Opin Struct Biol.

[15] Biertumpfel C, Basquin J, Suck D (2005). Practical implementations for improving the throughput in a manual crystallization setup. J Appl Cryst.

[16] Price WN, Chen Y, Handelman SK, Neely H, Manor P, Karlin R, Nair R, Liu J, Baran M, Everett J, Tong SN, Forouhar F, Swaminathan SS, Acton T, Xiao R, Luft JR, Lauricella A, DeTitta GT, Rost B, Montelione GT, Hunt JF (2009). Understanding the physical properties that control protein crystallization by analysis of large-scale experimental data. Nat Biotech.

[17] Canaves JM, Page R, Wilson IA, Stevens RA (2004). Protein biophysical properties that correlate with crystallisation success in *Thermotoga maritima*: maximum clustering strategy for structural genomics. J Mol Biol.

[18] Goh C, Lan N, Douglas S, Wu B, Echols N, Smith A, Milburn D, Montelione GT, Zhao H, Gerstein M (2004). Mining the structural genomics pipeline: identification of protein properties that affect high-throughput experimental analyses. J Mol Biol.

[19] Overton IM, Barton GJ (2006). A normalised scale for structural genomics target ranking: the OB-Score. FEBS Lett.

[20] Slabinski L, Jaroszewski L, Rychlewski L, Wilson IA, Lesley SA, Godzik A (2007). XtalPred: a web server for prediction of protein crystallizability. Bioinformatics.

[21] Overton IM, Padovani G, Girolami M, Barton GJ (2008). ParCrys: a Parzen window density estimation approach to protein crystallisation propensity prediction. Bioinformatics.

[22] Derewenda ZS, Vekilov PG (2006). Entropy and surface engineering in protein crystallization. Acta Crystallogr D Biol Crystallogr.

[23] Berman H, Henrick K, Nakamura H, Markley JL (2007). The worldwide Protein Data Bank (wwPDB): ensuring a single, uniform archive of PDB data. Nucl Acids Res.

[24] Baldi P, Brunak S, Dietterich T (1998). Bioinformatics: the machine learning approach.

[25] Andreeva A, Howorth D, Chandonia J-M, Brenner SE, Hubbard TJP, Chothia C, Murzin AG (2008). Data growth and its impact on the SCOP database: new developments. Nucl Acids Res.

[26] Chandonia JM, Hon G, Walker NS, Lo Conte L, Koehl P, Levitt M, Brenner SE (2004). The ASTRAL compendium in 2004. Nucleic Acids Res.

[27] Altschul SF, Madden TL, Schaffer AA, Zhang J, Zhang Z, Miller W, Lipman DJ (1997). Gapped BLAST and PSI-BLAST: a new generation of protein database search programs. Nucl Acids Res.

[28] Wootton, Federhen S (1996). Analysis of compositionally biased regions in sequence databases. Methods Enzymol.

[29] Apweiler R, Bairoch A, Wu CH, Barker WC, Boeckmann B, Ferro S, Gasteiger E, Huang H, Lopez R, Magrane M, Martin MJ, Natale DA, O'Donovan C, Redaschi N, Yeh LS (2004). UniProt: the Universal Protein Knowledgebase. Nucleic Acids Res.

[30] Rost B (1999). Twilight zone of protein sequence alignments. Protein Eng.

[31] Barton GJ, Sternberg M (1987). A strategy for the rapid multiple alignment of protein sequences: confidence levels from tertiary structure comparisons. J Mol Biol.

[32] Sonnhammer EL, Eddy SR, Birney E, Bateman A, Durbin R (1998). Pfam: multiple sequence alignments and HMM-profiles of protein domains. Nucl Acids Res.

[33] Bateman A, Coin L, Durbin R, Finn RD, Hollich V, Griffiths-Jones S, Khanna A, Marshall M, Moxon S, Sonnhammer ELL, Studholme D, Yeats C, Eddy SR (2004). The Pfam Protein Families Database. Nucleic Acids Res.

[34] Engelman DM, Steitz TA, Goldman A (1986). Identifying nonpolar transbilayer helices in amino acid sequences of membrane proteins. Annu Rev Biophys Biophys Chem.

[35] Cole C, Barber JD, Barton GJ (2008). The Jpred 3 secondary structure prediction server. Nucleic Acids Res.

[36] Yang ZR, Thomson R, McNeil P, Esnouf RM (2005). RONN: the bio-basis function neural network technique applied to the detection of natively disordered regions in proteins. Bioinformatics.

[37] Krogh A, Larsson B, von Heijne G, Sonnhammer ELL (2001). Predicting transmembrane protein topology with a hidden markov model: application to complete genomes. J Mol Biol.

[38] Smialowski P, Schmidt T, Cox J, Kirschner A, Frishman D (2006). Will my protein crystallize? A sequence-based predictor. Proteins.

[39] Bertone P, Kluger Y, Lan N, Zheng D, Christendat D, Yee A, Edwards AM, Arrowsmith CH, Montelione GT, Gerstein M (2001). SPINE: an integrated tracking database and data mining approach for identifying feasible targets in high-throughput structural proteomics. Nucl Acids Res.

[40] Christendat D, Yee A, Dharamsi A, Kluger Y, Savchenko A, Cort JR, Booth V, Mackereth CD, Saridakis V, Ekiel I, Kozlov G, Maxwell KL, Wu N, McIntosh LP, Gehring K, Kennedy MA, Davidson AR, Pai EF, Gerstein M, Edwards AM, Arrowsmith CH (2000). Structural proteomics of an archaeon. Nat Struct Mol Biol.

[41] Zell A, Mamier G, Vogt M, Mache N, Hubner R, Doring S, Herrmann K (1995). The SNNS users manual, version 4.1..

[42] R Development Core Team (2004). R: a language and environment for statistical computing.

[43] Bray JE, Marsden RL, Rison SCG, Savchenko A, Edwards AM, Thornton JM, Orengo CA (2004). A practical and robust sequence search strategy for structural genomics target selection. Bioinformatics.

[44] Chen L, Oughtred R, Berman HM, Westbrook J (2004). TargetDB: a target registration database for structural genomics projects. Bioinformatics.

[45] Chen C-N, Chu C-C, Zentella R, Pan S-M, David Ho T-H (2002). AtHVA22 gene family in Arabidopsis: phylogenetic relationship, ABA and stress regulation, and tissue-specific expression. Plant Mol Biol.

[46] Geeta L, Steven G (2000). Familial adenomatous polyposis. Semin Surg Oncol.

