# Supplementary material for: XANNpred: Neural nets that predict the propensity of a protein to yield diffraction-quality crystals
Source: Proteins. 2010 Oct 19;79(4):1027–33. doi: 10.1002/prot.22914 (PMC3084997; doi:10.1002/prot.22914)
Supplement: Supplementary file 1 [file prot0079-1027-SD1.pdf]

Supplementary Material for XANNpred: Neural Nets that Predict the Propensity of a Protein to Yield Diffraction-quality Crystals

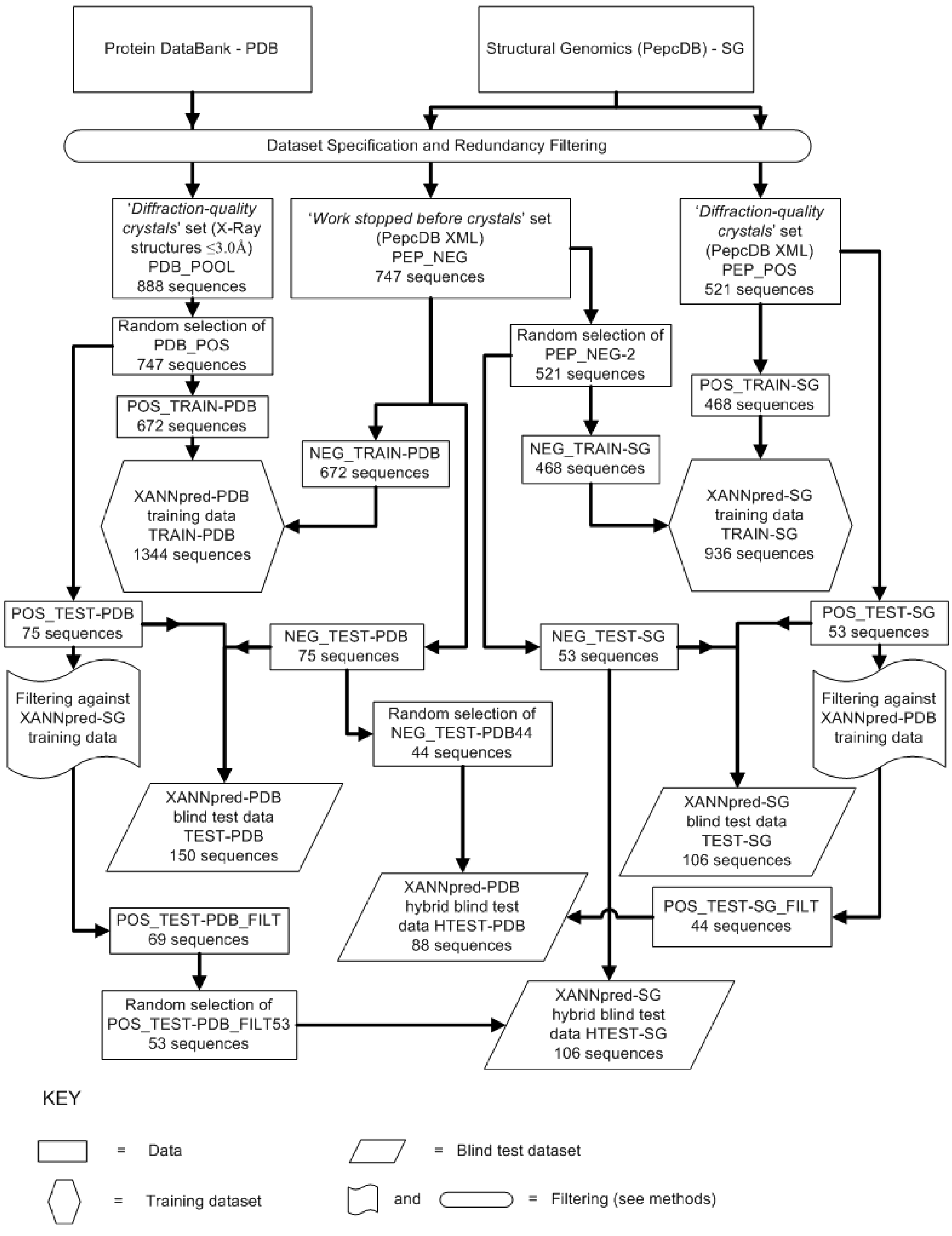

Figure S1. Summary of relationships between the datasets and their use in training and testing the algorithms XANNpred-PDB and XANNpred-SG. More details of individual datasets are given in Table S1 and in the main manuscript (Methods).

Section S1: Additional Details on XANNpred-SG Datasets

1.1 Balanced Training and Test Datasets

This section provides additional detail to that given in the main manuscript Methods & Materials section. In order to generate balanced datasets for training and testing XANNpred-SG, 521 sequences (PEP_NEG-2) were randomly selected from PEP_NEG to balance with the 521 sequences in PEP_POS. A random selection of 53 sequences from each of PEP_POS and PEP_NEG-2 were set aside as the blind test set (TEST-SG, 106 sequences). The remaining 468 sequences from each of PEP_POS and PEP_NEG (POS_TRAIN-SG and NEG_TRAIN-SG respectively) were combined to form the XANNpred-SG training dataset (TRAIN-SG, 936 sequences), which was input for 10-fold cross-validation.

1.2 Hybrid Test Datasets

This section provides additional detail to that given in the main manuscript Methods and Materials section. The 75 PDB sequences in TEST-PDB (POS_TEST-PDB) were searched against the XANNpred-SG training data with BLASTP[29]. Matches were assigned with published thresholds[32], and matching sequences were excluded to give POS_TEST-PDB_FILT (69 sequences). From POS_TEST-PDB_FILT 53 sequences were randomly selected (POS_TEST-PDB_FILT53) and combined with the 'work stopped' portion of TEST-SG (NEG_TEST-SG) to form the HTEST-SG dataset (106 sequences).

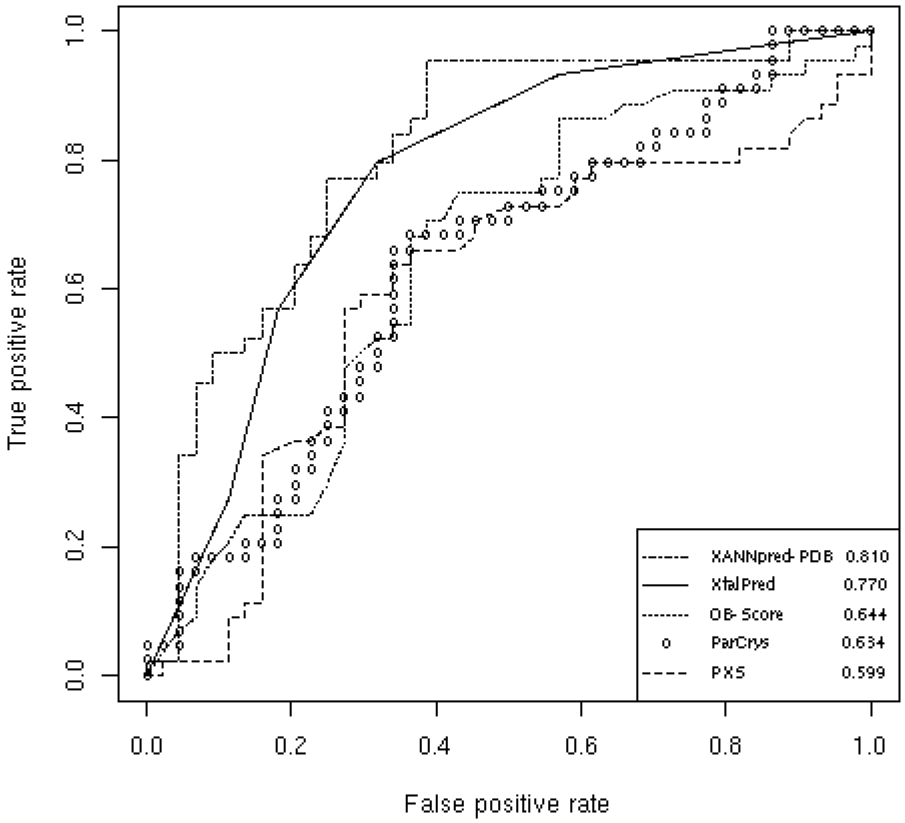

Figure S2. Performance over Hybrid Blind Test Dataset HTEST-PDB.
Receiver Operator Characteristic (ROC) curves for XANNpred-PDB, XtalPred, OB-Score, PXS and ParCrys over HTEST-PDB. Areas under the ROC curves are given in the bottom right-hand corner. This figure was generated using the R package[48].

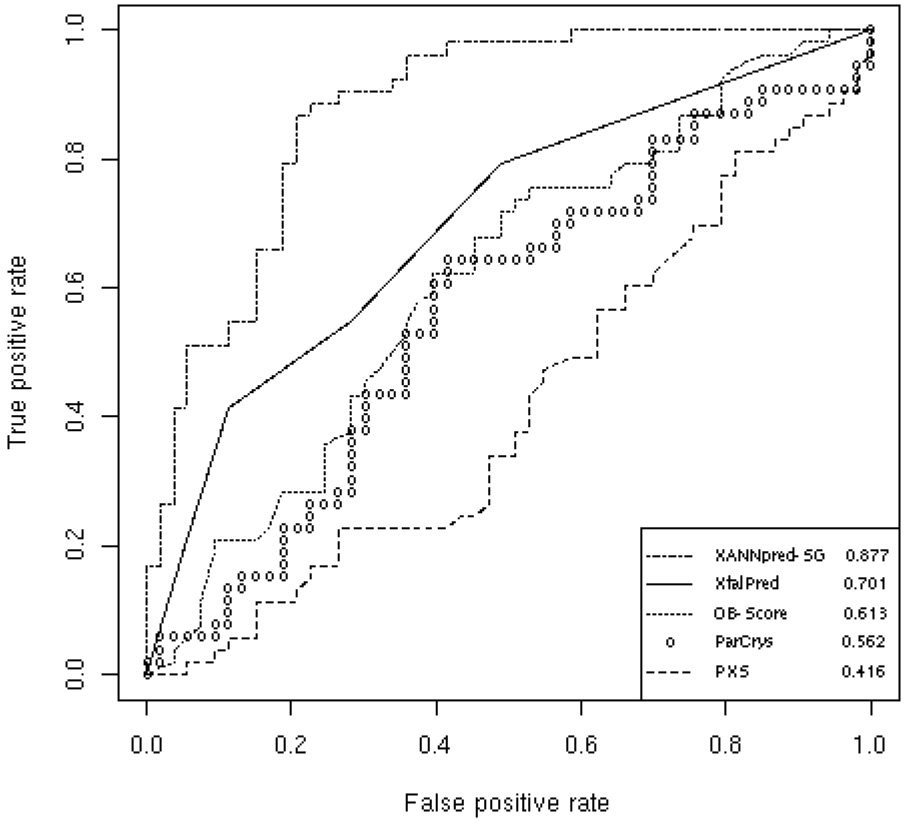

Figure S3. Performance over Hybrid Blind Test Dataset HTEST-SG.
Receiver Operator Characteristic (ROC) curves for XANNpred-SG XtalPred, OB-Score, PXS and ParCrys over HTEST-SG. Areas under the ROC curves are given in the bottom right-hand corner. This figure was generated using the R package[48].

Table S1 Summary of Datasets

Combined Dataset	Description	Dataset Name	Description	Size
TRAIN-PDB	XANNpred-PDB training dataset with ‘diffraction-quality crystals’ data from PDB and ‘work stopped’ data from PepcDB	POS_TRAIN-PDB	Positive training dataset based on PDB sequences	672
		NEG_TRAIN-PDB	Negative training dataset based on PepcDB sequences	672
TEST-PDB	XANNpred-PDB blind test dataset with ‘diffraction-quality crystals’ data from PDB and ‘work stopped’ data from PepcDB	POS_TEST-PDB	Positive blind test dataset based on PDB sequences	75
		NEG_TEST-PDB	Negative blind test dataset based on PepcDB sequences	75
TRAIN-SG	XANNpred-SG training dataset with both ‘diffraction-quality crystals’ and ‘work stopped’ data from PepcDB	POS_TRAIN-SG	Positive training dataset based on PepcDB sequences	468
		NEG_TRAIN-SG	Negative training dataset based on PepcDB sequences	468
TEST-SG	XANNpred-SG blind test dataset with both ‘diffraction-quality crystals’ and ‘work stopped’ data from PepcDB	POS_TEST-SG	Positive blind test dataset based on PepcDB sequences	53
		NEG_TEST-SG	Negative blind test set based on PepcDB sequences	53
PDB_POOL	Pool of redundancy-filtered PDB sequences	-	-	888
PDB_POS	Master positive dataset based on PDB, for XANNpred-PDB	POS_TRAIN-PDB	Positive training dataset based on PDB sequences	672
		POS_TEST-PDB	Positive blind test dataset based on PDB sequences	75
PEP_NEG	Master negative dataset based on PepcDB, for XANNpred-PDB	NEG_TRAIN-PDB	Negative training dataset based on PepcDB sequences	672
		NEG_TEST-PDB	Negative blind test dataset based on PepcDB sequences	75
PEP_POS	Master positive dataset based on PepcDB, for XANNpred-SG	POS_TRAIN-SG	Positive training dataset based on PepcDB sequences	468
		POS_TEST-SG	Positive blind test dataset based on PepcDB sequences	53
PEP_NEG-2	Master negative dataset based on PepcDB, for XANNpred-SG	NEG_TRAIN-SG	Negative training dataset based on PepcDB sequences	468
		NEG_TEST-SG	Negative blind test dataset based on PepcDB sequences	53
POS_TEST-PDB_FILT	Sequences from POS_TEST-PDB remaining after filtering against the XANNpred-SG training data. Used as a pool for POS_TEST-PDB_FILT53	-	-	69
HTEST-PDB	‘Hybrid’ blind test data for XANNpred-PDB with‘diffraction-quality crystals’ and ‘work stopped’ data from PepcDB	POS_TEST-SG_FILT	Positive blind test set based on PepcDB sequences and filtered against XANNpred-PDB training data	44
		NEG_TEST-PDB44	Negative blind test dataset based on PepcDB sequences	44
HTEST-SG	‘Hybrid’ blind test data for XANNpred-SG with‘diffraction-quality crystals’ data from PDB and ‘work stopped’ data from PepcDB	POS_TEST-PDB_FILT53	Positive blind test set based on PDB sequences and filtered against XANNpred-SG training data	53
		NEG_TEST-SG	Negative blind test set based on PepcDB sequences	53

Section S2: Feature Scaling

In order for the data to be presented to the neural network, each feature was scaled. Table S2 summarises the features chosen and ranges used for scaling. Scaling was done according to Equation 1 for all features except for the amino acid and dipeptide frequencies.

$$S = (r-m)/(x-m) \qquad (1)$$

Where:
S is the scaled parameter value
r is the raw parameter value
m is the minimum observed parameter value in the training data
x is the maximum observed parameter value in the training data

Table S2: Summary of Features

Feature	Min	Max
Fraction Jpred Helix	0	0.8
Fraction Jpred Strand	0	0.4
Fraction RONN disorder	0	0.6
Fraction TMHMM2 transmembrane regions	0	0.2
Average GES hydrophobicity	-1	1.0
Isoelectric Point	4.0	12
Sequence Length	0	800
Molecular Weight	0	80000
20 Amino acid frequencies	0	1
400 Dipeptide frequencies	0	1

Section S3: Conversion Of XtalPred Classes for Receiver Operator Characteristic Analysis

For the purpose of Receiver Operator Characteristic (ROC) analysis, the five XtalPred classes were translated into scores on a one to five scale where the most crystallisable ‘Optimal’ class scored 5 through to the least crystallisable ‘Very difficult’ class that had a score of one.
